# Exploring Professionals’ Perceptions of the Potential of Digital Twins in Homecare—A Focus Group Study in Sweden

**DOI:** 10.3390/healthcare14030289

**Published:** 2026-01-23

**Authors:** Sandra Saade, Susanna Nordin, Johan Borg

**Affiliations:** School of Health and Welfare, Dalarna University, SE-791 88 Falun, Sweden; snr@du.se (S.N.); jog@du.se (J.B.)

**Keywords:** caregiver support, digital health technology, digital twin, focus group, homecare, older adult, smart home technology

## Abstract

**Background/Objectives**: The growing number of older adults with complex healthcare needs increases demand for homecare services, while a shrinking workforce often lacks skills for advanced tasks. Digital health is seen as a promising tool to address these challenges. This study explored Swedish homecare professionals’ perceptions of the potential use of digital twins in daily work. **Methods**: Four focus group discussions were conducted with 31 homecare professionals; two groups each in one urban/rural and one rural municipality. Data were analyzed using inductive content analysis. **Results**: Three main themes emerged: (i) Perceptions of digital twins as support for older adults, including needs-based, individualized care and proactive support enabling preventive measures; (ii) Perceptions of digital twins as support for professionals, including a better work environment through streamlined tasks and flows and enhanced planning and assessment; and (iii) Concerns about digital twins, focusing on ethical and social issues and limited understanding, which were related to monitoring aspects, the importance of physical visits, and how the technology works. **Conclusions**: Digital twins are perceived by professionals to have the potential to improve homecare services by supporting both older adults and professionals; however, further research is needed to address concerns and practical implications.

## 1. Introduction

A global demographic shift is underway, characterized by an increasing proportion of older individuals and a declining working-age population [1]. More older adults with complex health conditions are living at home [2], due to ageing-in-place policies [3,4]. These individuals often have complex needs and frail health [5,6], and therefore require more assistance at home [7,8]. As part of this development, healthcare systems are facing a growing shortage of staff [9,10,11].

Digital health is an umbrella term for various technologies used to improve health [12]. It is increasingly seen as a promising tool for promoting healthy ageing and addressing challenges faced by health systems, such as demographic change [13,14]. Digital twins surpass conventional smart technologies by creating real-time virtual representations of physical systems, enabling predictive analysis, simulation, and adaptive automation. Unlike other smart technology solutions that focus on connectivity and monitoring, digital twins provide a dynamic, bidirectional link between physical and digital environments, driving intelligent decision-making and user-centric optimization [15]. Research on digital twins within healthcare has grown rapidly in recent years, but remains at an early stage, primarily at a conceptual level [16]. Digital twins are applied to develop virtual models of patients, healthcare settings, and medical equipment, resulting in more personalized care [17]. Digital twins are applied to personalize treatment, optimize care, support real-time decision-making, enhance operational efficiency and resource management, and drive innovation in medical research [18]. They can offer training opportunities to professionals, and be used proactively to prevent accidents, streamline workflows, and enhance patient outcomes and safety [19]. There is, however, a lack of implemented digital twins in healthcare [20,21], and studies on digital twins within homecare are scarce [22]. Unresolved questions remain regarding technical aspects, data-related concerns, and ethical challenges [16,23,24].

Homecare can include medical care services [25], but in Sweden, homecare is practical and personal support at home, addressing physical, psychological, and social needs, and may involve help with hygiene, dressing, eating, and household tasks [26]. The municipalities in Sweden are responsible for providing homecare, which is regulated by the Social Services Act [27]. Homecare is provided by homecare workers, of whom most are licenced practical nurses, which is the most prevalent occupation in Sweden [28], however staff continuity is generally low [29]. They are expected to perform more advanced tasks [30] and promote autonomy, dignity, and well-being through individualized, preventive care [27]. However, they may lack the necessary competencies and require additional knowledge to meet the complex needs of older adults [31,32,33]. Moreover, high workloads contribute to uncertainty about care quality and concerns about exposing older adults to risks [34]. Homecare workers may also face physical risks, such as musculoskeletal injuries [35], and psychosocial risks related to stress from organizational issues like overtime and lack of managerial support [36]. However, digital health technologies can improve care delivery and clinical decision-making among healthcare workers [9,37].

Integrated care services are increasingly recognized as a global and national reform to strengthen health systems in response to demographic change, emphasizing person-centred and coordinated care with a focus on community care, and where digital technologies are encouraged [38,39,40]. Person-centred care is grounded in values such as respect for personhood, authenticity, shared decision-making, mutual respect, partnership, and therapeutic relationships [41]. Digital health technologies can support preventive care, reduce costs, and increase accessibility, factors that are central to the implementation of integrated care [42]. Despite the substantial benefits that the reform can bring, a recent report from Sweden shows that the objectives of the integrated care reform remain unmet [43].

More research on digital health technologies, with diverse study designs and including the perspectives of caregivers, is needed [44]. Recognizing how digital twins can be used in professionals’ daily work is essential, as research demonstrates that the well-being and work-related perceptions of both homecare professionals and managers influence the quality of care [45,46,47]. Understanding how homecare professionals perceive and potentially adopt digital twins is also essential and requires attention to the factors that influence technology acceptance. The Unified Theory of Acceptance and Use of Technology (UTAUT) [48] provides a theoretical framework for explaining how individuals adopt new technologies. It identifies four key constructs that influence behavioural intention and actual use: performance expectancy, effort expectancy, social influence, and facilitating conditions. Applying UTAUT to homecare can help identify the conditions under which digital twins might be perceived as useful, easy to use, and supported by organizational structures, while also considering social norms.

As demographic shifts and policy changes continue to reshape homecare, digital health technologies are seen as promising tools to support integrated, person-centred care [42,49,50,51]. Among these, digital twins hold potential to address challenges related to demographic change. However, to our knowledge, no studies have explored the perspective of professionals in relation to digital twins in homecare. Gaining such insights would be crucial for the development of digital twin solutions, particularly given that staff continuity is low and many professionals lack sufficient competence and knowledge to meet the needs of older adults. Therefore, this study aimed to explore how homecare professionals in Sweden perceive the potential use of digital twins in their daily work.

## 2. Materials and Methods

### 2.1. Design

This study employed an inductive qualitative approach, with data collected through semi-structured focus group discussions. Considering the limited existing knowledge on digital twins in homecare, an inductive approach was applied [52], enabling the development of general patterns and insights from empirical data [53]. To ensure comprehensive and transparent reporting of the findings, the checklist of consolidated criteria for reporting qualitative research (COREQ) was used [54] and is included in the Supplementary Materials.

### 2.2. Research Team and Reflexivity

The research team holds diverse professional backgrounds; the first and second authors are both females and registered nurses. The first author is a doctoral student, and the second author holds a PhD, and has extensive experience in qualitative research. The third author is male, holds a PhD, and has expertise in digital health technologies. Drawing on personal and interpersonal reflexivity [55], the authors engaged in continuous reflection throughout the study. Personal reflexivity involved critically examining our assumptions, prior experiences, and professional identities, acknowledging how these could shape decisions and interpretations. Given that the research team has an interest and works with technologies, the researchers were particularly attentive to the possibility of holding overly positive views of such technologies and sought to ensure that these perspectives did not unduly influence the analysis or interpretation of findings. Interpersonal reflexivity focused on the relationships within the research team and between researchers and participants. We considered how our professional background and positions might create power dynamics and influence participants’ responses. To mitigate these influences, we engaged in regular team meetings and discussions to explore differing interpretations and disagreements.

### 2.3. Participants and Settings

Participants were recruited from a county located in central Sweden, with one of the highest average ages in Sweden [56]. Two municipalities were included: one with approximately 60,000 inhabitants, classified as an urban/rural municipality, and the other with approximately 11,000 inhabitants, classified as a rural municipality [57]. A unit manager for the homecare services assisted with recruitment in one municipality, while a social services system administrator did so in the other. Both recruiters supported the study by inviting professionals from their respective workplaces to participate in a focus group. A purposive sample strategy was employed. The inclusion criteria required participants to hold a permanent position within homecare services and represent a variety of professional roles.

Four face-to-face focus group discussions were conducted (two in each municipality), which aligns with the number usually required to achieve data saturation in qualitative research [58]. Saturation was assessed during data collection and analysis and was considered achieved when no new patterns emerged in the final group discussion. This was confirmed by the researchers’ experience and ongoing comparison of data across groups. Totally, 31 individuals (22 women and 9 men) participated in the four focus group discussions. Characteristics of the participants are presented in Table 1.

The professionals included homecare workers (working day and night shifts; n = 9) who deliver care services to older adults, care planners (n = 4) who manage scheduling of homecare workers, and case managers (n = 3) who assess individuals’ needs and decide on the allocation of homecare services. Unit managers (n = 5) oversee daily operations, staff management, and quality of care, ensuring service delivery aligns with municipal guidelines. Operations managers (n = 3) coordinate and supervise municipal care services, IT specialists/system administrators (n = 4) maintain digital infrastructure, support technology implementation, ensure data security, and provide technical assistance to staff, and technicians (n = 3) work with homecare-related technologies such as personal alarms.

### 2.4. Data Collection

Focus group discussions were chosen as they are an efficient method for collecting rich, in-depth data within a short time [53]. The focus group discussions took place between September and November 2023. Two focus groups were conducted at a university, conveniently located for those participants, and were moderated by the first and second authors, while the other two were held in a centrally located municipal venue to facilitate participation for individuals living in that area, and were moderated by the second and third authors.

During the sessions, a set of predetermined, semi-structured questions guided the discussions. First, ground rules were introduced, emphasizing that all perspectives were welcome, and that confidentiality would be maintained. Second, a brief overview of what a digital twin is and how it works was presented. None of the participants possessed in-depth knowledge of digital twins, nor had they had any prior experience using them; consequently, their responses about digital twins were hypothetical in nature. The group discussions began by allowing participants to reflect on their work experiences and what they need to know to carry out their tasks, providing valuable insights for the researchers into the structure and functioning of the homecare system and helping them to better understand the participants’ thoughts about digital twins. The main question during the discussions was: “How would you like a digital twin to help you in your work?” (see Appendix A). Each group discussion lasted approximately 65 min on average, with all extending beyond one hour. The focus groups were audio-recorded and transcribed verbatim, a professional transcriber completed the majority of the transcripts, while the first author transcribed one. Given the limited existing knowledge on digital twins in homecare, the researchers adhered strictly to the recorded material to ensure that the analysis was grounded in participants’ expressed perspectives.

### 2.5. Data Analysis

Data was analyzed using qualitative content analysis [52]. This approach can uncover new perspectives, deepen researchers’ understanding of a topic, and inform practical actions [59]. To become familiar with the material, the transcribed data were read several times, allowing key concepts to emerge [53]. The text was closely followed to ensure that the findings were grounded in the participants’ own words.

After becoming immersed in the text, meaning units, which could be a sentence or more from the transcribed text, and related to the aim of this study, were identified. The meaning units were then coded. The codes were one or a few words describing the meaning unit. After this step, the codes were grouped under higher order headings and grouped into categories and themes.

The analysis followed an iterative process, involving movement between the different analysis steps. The first author was responsible for the analysis, but all the authors repeatedly got together and discussed the meaning units, codes, categories, and themes until agreement was reached, thereby enhancing the trustworthiness of the study [60]. When disagreements occurred, they were resolved through critical dialogue and re-examination of the data, by revisiting original transcripts to ensure shared interpretation. Quotations were used to enhance the credibility of the analysis. Table 2 shows an example of the analysis process.

### 2.6. Ethical Considerations

The ethical principles of the Declaration of Helsinki were followed throughout the study [61]. An approval from the Swedish Ethical Review Authority (2023-04272-01) was obtained. Written and oral consents were obtained from all participants, who were also informed of their right to withdraw from the study at any time.

## 3. Results

Three overarching themes emerged from the analysis, with two categories identified within each theme (see Table 3).

### 3.1. Perceptions of Digital Twins as Support for Older Adults

Participants frequently described how digital twins could potentially enhance support for older adults, emphasizing perceived benefits. While they outlined a range of possible practical applications, these reflect expectations rather than implemented solutions. They consistently emphasized the importance of tailoring support to the individual needs and preferences of older adults.

Two anticipated forms of support emerged: *needs-based support*, which responds to specific and immediate requirements, and *proactive support*, which anticipates potential needs before they arise.

Participants underscored the importance of addressing the specific needs of older adults and suggested that digital twins might contribute to more needs-based support. For instance, participants envisioned how digital twins could lead to faster identification of needs. They also envisioned how digital twins could enhance the timing and relevance of homecare services by monitoring circadian rhythms and daily activity patterns such as sleep, eating, and toileting routines. While acknowledging that care should ideally follow the individual’s needs and preferences, in practice, visits were often driven by rigid schedules, not actual needs. This mismatch sometimes resulted in older adults being woken maturely or receiving support at inopportune times. The following example illustrates participants’ expectations that digital twins could help align visits with actual needs, occurring at the appropriate time and moment.

*“Like, that we might be there at the right time. That we’re not there, and then the older adult has already eaten. Or we were there, and no, they didn’t want to [eat], and then you’re there afterwards. Or when they need to go to the toilet and you’re not there at the right time. More like seeing that you time it in a good way.”*

(Woman, focus group A)

Participants also saw potential for redistributing time more effectively. By reducing unnecessary or mis-timed homecare services, more attention could be given to activities that are often neglected due to time constraints.

*“But if certain services can be reduced, maybe we could increase those things that we normally don’t really have time for, like giving them physical activity, I mean going for walks with them, which often there isn’t time for, and stuff like that.”*

(Woman, focus group D)

Participants anticipated that digital twins could enable professionals to gain insight into the home environment (e.g., temperature) and the older adults’ activities or acute incidents (e.g., falls), even when not physically present. These perceived benefits were considered helpful for preparing and tailoring visits more closely to the individual’s needs.

According to participants, digital twins could also offer proactive support by enabling the anticipation of events and situations, thereby facilitating proactive measures and reducing the risk of adverse incidents. Proactive measures could lead to time being alleviated, and include preventing accidents, limiting the spread of infections, and initiating targeted activities to improve situations before they deteriorate, such as addressing falls or deviations in health. Participants further envisioned that the ability of digital twins to predict changes that could be made to the physical home environment was considered to have the potential to promote independence.

*“So that you know, if, maybe other assistive products are needed, or if rehab needs to be contacted…if you somehow register that someone walks into a doorframe with their walker or something like that…then you could easily get a notification like, yes assistive products are needed…if it’s necessary to make a housing adaptation or replace something…or if more services are needed.”*

(Woman, focus group B)

### 3.2. Perceptions of Digital Twins as Support for Professionals

Participants described that digital twins could potentially support the professionals themselves. This anticipated support could be more targeted at the homecare professionals who carry out homecare visits or the ones working with planning, staff management, and assessment of homecare services. This resulted in two categories targeting two different kinds of support: *better work environment* and *planning and assessment*.

Digital twins were described as having the potential to improve the work environment by streamlining tasks and supporting staff in their daily routines. The participants emphasized that such technology could create more efficient workflows and reduce unnecessary steps. They also highlighted the value of real-time functions, such as alerting professionals in threatening situations or when mobile phone coverage was unreliable, making communication difficult. Examples were given of situations where staff visited an older person at home only to discover that the person had already been admitted to hospital care. These visits were often made “just in case,” but ultimately resulted in avoidable travel and time loss, resources that could have been allocated more effectively. It was suggested that a digital twin could help prevent such scenarios by providing up-to-date information. Participants further envisioned digital twins as a means of reducing stress, thereby lowering the risk of errors or deviations in care. As one participant explained:

*“If time is limited, then you could have carried out other interventions at a calmer pace. Not risk making a mistake because you are stressed, and then you go on to the next visit and you are still stressed, then there can be deviations in older adults’ interventions.”*

(Man, focus group A)

Additionally, digital twins were viewed as a way to facilitate professionals’ workflows by helping to prevent falls and injuries, as mentioned in the paragraph above from the user’s point of view. The benefits extended to the professionals as well; participants noted that when an older adult required less assistance, visits could be simpler compared to situations where e.g., a fall had occurred, requiring more services and double staffing.

Participants considered digital twins to have potential in supporting planning and assessment, including professionals’ scheduling, time planning of services, and assessment of homecare services. Regarding scheduling, digital twins could work as tools for simulating outcomes of changes made within the schedule, allowing users to visualize the effects of a single adjustment. This capability was particularly valued by those responsible for planning, as it enables them to identify where further modifications might be needed. Additionally, participants suggested that by reflecting the actual time required for specific care tasks, digital twins might contribute to more accurate time planning.

*“Because it is so complex sometimes, you know that as the one doing the planning. You don’t really know what the end result will be. If you make a change in one place, you have to adjust in many other places. And if you then have a data tool that helps with that, you can do it much faster. And then you can get outcomes without having to do it in reality, so to speak, I think. That would be a great help.”*

(Man, focus group C)

Participants considered digital twins to have potential as tools for planning driving routes and estimating travel times when cars were used, which would facilitate overall scheduling. Digital twins were also perceived as useful for evaluating whether appropriate care interventions had been assigned, for prioritizing, and as a help when doing a follow-up to assess whether the planned services align with the identified needs. For example, participants noted that in cases of uncertainty, it was common to initially assign a high number of homecare visits, which might later be reduced. With the help of digital twins, such over-allocation could potentially be avoided.

*“For me as a manager, this could really help a lot in understanding what staffing needs we actually have throughout the day.”*

(Man, focus group C)

Digital twins were also envisioned as a tool that might provide a clearer picture of actual needs and serve as valuable support in situations where perspectives differ, such as between family members and case managers, or when there is a discrepancy between the older adult’s perceived needs and those identified by the case manager, thereby enabling more accurate assessments.

### 3.3. Concerns About Digital Twins

Participants expressed concerns regarding digital twins, including *ethical and social issues*, and a general *lack of understanding* about what a digital twin is and how it can be utilized.

They questioned whether monitoring individuals in this way would be acceptable, leading to ethical and social issues. Discussions included concerns about older adults being monitored and whether it would be appropriate for the professionals themselves to be monitored.

*“You mention that you want to support the staff and also track their patterns, have you addressed how you plan to negotiate with the union about this?”*

(Man, focus group D)

There were also reflections on the importance of physical encounters for older adults. Participants emphasized that visits are vital for social interaction and cannot be fully replaced by other means. They often believed that in-person meetings are crucial for older adults, and that the use of digital twins might reduce the frequency of such visits. At the same time, some participants considered that digital twins, functioning as a form of remote supervision, could be beneficial for older individuals who receive too many visits and feel that they lack privacy.

*“Well, I think these check-ins are good, for example, if you have a camera for a visit. But you also need physical contact, it is important.”*

(Woman, focus group B)

Some concerns were also raised in relation to the lack of understanding of digital twins and questions regarding how the technology should be used in practice. Participants often struggled to provide concrete examples of how digital twins could support them due to difficulties in understanding how the technology could function in practice.

*“I’m thinking, how are you going to collect all the data? I mean, are you going to install sensors in users’ homes or what?”*

(Woman, focus group B)

The participants anticipated that digital twins could potentially increase administrative burdens for professionals, as they would be responsible for monitoring and responding to events in real-time. Questions were raised about whether the digital twins would need to be continuously updated, and how such updates would be managed. The professionals also noted that current legislation does not appear to align with the technological developments.

## 4. Discussion

This study explored professionals’ perspectives on the potential use of digital twins in homecare, aiming to understand how they perceive the technology’s role in daily work tasks. The main findings showed that professionals believe digital twins could support both older adults and staff, but they also reported limited understanding of the technology and raised concerns about ethical and social implications. The findings support the potential of digital twins to enable more person-centred homecare that is consistent with the routines, daily activities, preferences, and needs of older adults. These findings are partly consistent with previous studies that emphasize the importance of tailoring care to the individual, including research on diabetes, dementia, and musculoskeletal diseases [62,63,64]. However, it is important to note that these studies have not been conducted within the context of homecare for older adults, and the majority have not been implemented in practice. The findings also support the potential of digital twins to lead to more proactive homecare, an aspect that has been highlighted in other studies [17,18,65], often in the context of preventive medicine aimed at treating diseases and medical symptoms. However, these studies addressed personalized care and preventive medicine to treat diseases and individualize treatments, which do not align with the focus of homecare in this study.

In our study, the professionals identified the potential for proactive support by anticipating future needs, e.g., rehabilitation, and initiating interventions before issues arise. Such proactive strategies may be particularly beneficial for older adults receiving homecare, as such approaches can enhance their sense of safety, as demonstrated in a study where sensor-based monitoring was implemented in the home environment [66]. To translate this potential into practice, initial steps could include digital twin simulations into existing homecare planning and scheduling systems. Selected municipalities could start with pilot programs on a small scale to test feasibility and inform guidelines for broader adoption.

The professionals not only envisioned digital twins as being used to improve homecare services from the perspective of older adults, but also highlighted the role of digital twins in enhancing the work environment and facilitating planning and assessment within homecare. Digital twin technology has the potential to support not only homecare workers but also managers and care planners responsible for daily operations and assessments. For care workers, digital twins may provide insight into conditions in the home prior to a visit, enabling better preparation and more responsive care. For managers and planners, the simulation capabilities of digital twins may support the visualization of potential outcomes without implementing changes in practice, thereby improving scheduling, as discussed in previous research [67,68,69], optimizing time allocation for visits, and supporting the evaluation of whether planned activities are appropriate and effective. Within the UTAUT framework [48], these perceived benefits correspond to performance expectancy, as professionals anticipated that digital twins could enhance work efficiency and care quality. Such expectations are likely to positively influence behavioural intention and, ultimately, the actual use of digital twin technology. However, participants also anticipated increased administrative burdens and expressed concerns regarding misalignment between existing legislation and technological developments. These concerns relate to effort expectancy, as additional workload may reduce perceived ease of use, as well as facilitating conditions, particularly in terms of legal, organizational, and technical infrastructure. To support future implementation and adoption of digital twins, it is therefore essential to address these factors by ensuring that professionals can easily interpret system outputs without additional workload, supported by a user-friendly interface and a reliable infrastructure tailored to their needs. Addressing challenges in the technology by addressing technical features ensures that the generated knowledge is accurate, trusted, and easy to interpret [70]. Likewise, supporting the persons adopting a new technology by avoiding additional workload, is essential for successful adoption and sustainability.

Since well-being and work-related perceptions of homecare workers and managers influence the quality of care [45,46,47], it is essential not to focus solely on how digital twins may benefit older adults, but also on how they may benefit professionals. The professionals working in the care of older adults in Sweden, are reported to be on sick leave twice as often and for twice as long as professionals in other occupations [28]. On the other hand, they report experiencing a high level of meaningfulness in their work [28,34], a finding that was reflected in the focus group discussions conducted in this study. Supporting and improving their working conditions may therefore not only enhance care quality, as previously noted, but also contribute to increased staff retention [71,72]. The persistently high turnover rates in care of older adults [73,74] could also be helped by this.

Time seems central for homecare professionals during their daily work. They noted that digital twins could help optimize time by enabling better planning and preventing health deterioration or accidents. Digital twins can help homecare workers to be on time and also to know how much time is needed, which helps to redistribute time more effectively. Homecare workers also reflected on the importance of having sufficient time during a visit, noting that sufficient time reduces stress. Both professionals and older adults appear to value time as a critical factor in homecare. It has been reported [75] that older adults emphasize the need for longer and more timely visits, identifying these as key elements in addressing unmet care needs. Additionally, time pressure was perceived negatively.

Concerns related to digital twins primarily evolved around ethical and social implications, as well as a general lack of understanding of what digital twins are and how they may function. Issues on privacy, integrity, and data security remain unsolved regarding digital twins [23,76,77], and may affect the acceptance of digital twins [78]. It is crucial to develop ethical guidelines for the application and management of digital twins [24] before the technology is widely deployed. Based on insights from UTAUT [48], potential barriers to adoption can be anticipated, enabling researchers to design digital health technologies that foster acceptance. Findings from this study indicate that there is still no broad understanding of digital twins, which could influence the different constructs of the framework, especially the construct of social influence, which refers to the degree to which an individual perceives that an important other thinks they should use a technology. To address this construct, efforts should focus on cultivating a sense of collective acceptance and shared norms surrounding technology adoption. Raising awareness of how digital twins may be used is essential for helping professionals recognize their benefits more clearly. Given these concerns raised about digital twins, effective implementations may still be some distance away, but they are crucial for advancing knowledge and building a robust evidence base on their potential benefits.

In sum, the findings show that digital twins are perceived to have the potential to lead to more proactive, person-centred, and coordinated care, which are key factors of integrated care [79], while digital health technologies are encouraged as part of achieving the integrated care reform [38,40]. The findings also show that homecare professionals may benefit from their potential in the form of a better work environment with less stress. However, some concerns need attention. It is important to integrate questions that address aspects of the technology, such as privacy, ethics, and data security, as well as aspects focusing on user perceptions and behavioural intentions when developing digital twins.

### Strengths and Limitations

By using a purposeful sampling to include professionals with diverse experiences and insights from homecare, a broader understanding of how digital twins could be used in homecare was achieved. The value of homogeneity in focus group composition is acknowledged [53], but in this study, the broader perspective, was intended and desirable. The diverse professionals’ perceptions captured the potential use of digital twins at several levels. However, the findings may hold a limitation since the participants had no direct experience with digital twins, and therefore, the findings are based on hypothetical perceptions.

Furthermore, the study was conducted in only two municipalities located in a Swedish region with one of the highest average ages in the population. This demographic context may have influenced participants’ heightened awareness of challenges affecting homecare services. While the findings may be particularly relevant to similar ageing contexts, caution is warranted when considering the transferability of the results to other settings. Municipalities of different sizes and sociodemographic compositions may face distinct needs and priorities, and variations in homecare organization and service delivery across countries should also be considered.

Credibility of the results was maintained throughout all stages of the study. Participants were thoroughly described, and data saturation was achieved. Several researchers were involved in data collection, with one attending all four groups. While one researcher led the analysis as suggested [60], regular meetings were held where all researchers engaged in discussions regarding categorization and iteratively went through the data to enhance credibility. The content analysis process is reported in detail, illustrating the steps of the analysis, which enhances dependability.

## 5. Conclusions

This study offers a novel contribution by exploring homecare professionals’ perspectives on the potential use of digital twins in homecare, a field where such technologies remain largely untested. Future research may benefit from moving beyond conceptual design and including studies investigating ethical considerations, user engagement, and the practical implications of digital twins. These insights could help inform the development of integrated solutions that are responsive to the evolving needs of older adults and the professionals who support them.

## Figures and Tables

**Table 1:** Characteristics of the participants.

Focus Groups	A	B	C	D
Number of participants ^1^	9	8	9	8
Male	2	2	4	1
Female	7	6	5	7
Age range (years)	27–62	34–58	32–59	44–64
(mean age)	(45)	(45)	(41)	(53)
Work experience in care for older adults in years (mean)	20	16	16	17
Work experience in homecare in years (mean)	11	15	12	13

^1^ Three participants participated in two focus groups, resulting in a total of 31 participating individuals.

**Table 2:** An example of the analysis process.

Meaning Units	Codes	Category	Theme
“Something that would have been helpful to facilitate would have been to map out how much time this client needs for each intervention.”	Facilitate schedule How much time is needed	Planning and assessment	Perceptions of digital twins as support for professionals
“So, you can easily see if the person in question is at home. I think there are many visits in a month that you make unnecessarily.”	Can see if the person is at home Unnecessary visits		

**Table 3:** Overview of the themes and categories.

Themes	Categories
Perceptions of digital twins as support for older adults	Needs-based support Proactive support
Perceptions of digital twins as support for professionals	Better work environment Planning and assessment
Concerns about digital twins	Ethical and social issues Lack of understanding

## Data Availability

Due to ethical considerations, the data from this study can not be shared publicly. Anonymized data that support the study findings are available from the corresponding author upon reasonable request.

## Supplementary Materials

The following supporting information can be downloaded at: https://www.mdpi.com/article/10.3390/healthcare14030289/s1. COREQ (COnsolidated criteria for REporting Qualitative research) Checklist.

## Appendix A

Questions and examples of prompts:

1. Most important and valuable aspects of working in homecare
  - What tasks that you perform are perceived as the most valuable/important?
  - Which tasks do you think are the most important for older adults?
  - What values are important?
2. You are many who work in homecare, and you have different roles…
  - What would you like to know about each other to do a good job? (e.g., where someone is/working hours/sickness among the staff)
  - What do you need to know to be able to plan the work?
  - How do you find out such things today?
  - What would you like to know about the daily lives of older adults to do a good job?
3. How would you like a digital twin to assist you in your work?
  - What expectations and wishes do you have for the functions of a digital twin?
  - What should it measure? What properties might be important to measure? What do you want to get out of it? What should it support?
